# Genomic Diversity, Virulome, and Resistome of *Streptococcus agalactiae* in Northeastern Brazil: Are Multi-Host Adapted Strains Rising?

**DOI:** 10.3390/pathogens14030292

**Published:** 2025-03-17

**Authors:** Vinicius Pietta Perez, Luciana Roberta Torini, Fernanda Zani Manieri, Suellen Bernardo de Queiroz, Jorhanna Isabelle Araujo de Brito Gomes, Lauro Santos Filho, Eloiza Helena Campana, Celso Jose Bruno de Oliveira, Eduardo Sergio Soares Sousa, Ilana Lopes Baratella Cunha Camargo

**Affiliations:** 1 Núcleo de Medicina Tropical—NUMETROP, Centro de Ciências da Saúde, Universidade Federal da Paraíba, Campus I, João Pessoa 58050-085, PB, Brazil; sbq@academico.ufpb.br (S.B.d.Q.); jorhanna.gomes@academico.ufpb.br (J.I.A.d.B.G.); lauro.ufpb@hotmail.com (L.S.F.); 2 Laboratório de Epidemiologia e Microbiologia Moleculares—LEMiMo, Instituto de Física de São Carlos, Universidade de São Paulo, São Carlos 13563-120, SP, Brazil; lucianatorini@usp.br (L.R.T.); fzmanieri@usp.br (F.Z.M.); 3 Laboratório de Biologia Molecular—LABIMOL, Centro de Ciências Médicas, Universidade Federal da Paraíba, Campus I, João Pessoa 58050-085, PB, Brazilesergiosousa@uol.com.br (E.S.S.S.); 4 Laboratório de Avaliação de Produtos de Origem Animal—LAPOA, Centro de Ciências Agrárias, Campus II, Areia 58397-000, PB, Brazil; celso.bruno.oliveira@gmail.com

**Keywords:** group B streptococci, GBS, capsular serotype, clonal complex, virulence, resistance, epidemiology

## Abstract

*Streptococcus agalactiae*, known as group B streptococci (GBS), colonizes the digestive and genitourinary tracts and causes neonatal diseases and infections in immunocompromised and elderly individuals. GBS neonatal disease prevention includes intrapartum antibiotic prophylaxis. We characterized 101 GBS isolates obtained from patients in João Pessoa, northeastern Brazil, owing to the need to develop and implement vaccines to prevent GBS infections. Capsular types were determined using multiplex-PCR, and antibiotic susceptibility profiles were determined using disc diffusion or the gradient strip method. Clonal diversity was evaluated using pulsed-field gel electrophoresis. Fourteen selected isolates had the genome sequenced and evaluated for virulence and resistance genes. The GBS population had high clonal diversity, with serotype Ia and V prevalence. Among the sequenced isolates, we detected antibiotic resistance genes (*ant(6)-Ia*, *catA8*, *ermA*, *ermB*, *lsaE*, *lsnuB*, *mefA*/*msrD*, *tetM*, *tetO*, and *tetS*), several virulence genes, and mobile genetic elements integrated into the chromosome. The most frequent Sequence Type (ST) was ST144, followed by ST196, ST28, ST19, ST12, ST23, ST103, and the new ST1983 (CC103). Phylogenetically, ST103 and ST1983 were distant from the other STs. Our data revealed highly virulent GBS strains in this population and a new ST that could be related to a zoonotic origin.

## 1. Introduction

*Streptococcus agalactiae*, also known as group B streptococci (GBS), are commensal members of the human microbiota. GBS is an opportunistic pathogen that causes infections in mothers, neonates, young infants, and immunocompromised patients [1].

Neonatal infections are associated with significant morbidity and mortality. Early onset infections (EOI) typically manifest within the first week of life and are primarily acquired during delivery. To prevent EOI, administering intrapartum antibiotic prophylaxis (IAP) during labor is recommended [2]. In contrast, late-onset infections (LOI) manifest during the first 3 months of life and are acquired from the mother or from environmental sources [3]. Prenatal-onset infections (POI) occur before birth and are often unrecognizable. Although there are no effective measures to prevent LOI and POI, vaccine development remains a promising strategy [2].

Several GBS virulence factors contribute to cell adhesion and immune evasion. The *bca* gene encodes surface protein C (alpha antigen), which promotes epithelial cell penetration, whereas the *bac* gene encodes surface protein C (beta antigen), which binds IgA antibodies and human factor H. *cspA* encodes a serine protease that cleaves fibrinogen and degrades chemokines, whereas *cylE* encodes cytolysin, which is involved in cell invasion and tissue damage.

The *fbsA*/*B*/*C* genes encode fibrinogen-binding proteins and *sip* encodes a surface immunogenic protein [4]. The *hylB* gene encodes hyaluronidase, which impairs neutrophil function [5], and *pbsP* encodes a plasminogen-binding protein essential for GBS pathogenesis [6]. The *srr* gene encodes glycoproteins with a serine-rich repeat domain, and *lmb* encodes a laminin-binding protein. Additionally, *scpB* encodes C5a peptidase, which inactivates complement factor C5a in the human host [7]. The expression of the HvgA protein enhances adherence to intestinal epithelial cells, the choroid plexus, and microvascular endothelial cells, and is found in the highly virulent ST17 [8]. Furthermore, persistent streptococcal colonization is associated with pili, which play a role in biofilm formation [9].

Current strategies to prevent GBS involve maternal and neonatal exposure to antibiotics, but several reports have indicated increased GBS antibiotic resistance [10,11,12,13]. Resistance to macrolides, mediated by the MefA/MsrD efflux pump or erythromycin methylases (*erm* genes), has prompted revisions to the IAP protocols [14]. Although beta-lactams remain the first-line therapy, GBS susceptibility to these antibiotics has shown variability worldwide. Its susceptibility to clindamycin, the second-line agent, needs to be confirmed because *erm* genes can also confer resistance to lincosamides, and isolates harboring *lsa* and *lnu* genes have been reported. Resistance to tetracyclines is a common characteristic of GBS strains and is primarily associated with ribosome protection proteins (TetM, TetO, TetS, and TetT) [10,12]. Continuous surveillance of GBS antibiotic resistance is essential to optimize IAP protocols and guide treatment strategies.

Epidemiological and genomic data of GBS isolated from patients in northeastern Brazil are limited. This study investigated the clonality, serotype distribution, antibiotic susceptibility, and virulence backgrounds of 101 GBS isolates. Additionally, taking advantage of whole-genome sequencing using short- and long-read sequencing methodologies of 14 isolates, we provide unique findings for the GBS population and a new sequence type (ST) description.

## 2. Materials and Methods

### 2.1. Strain Isolation and Identification

Isolates of different clinical specimens obtained from patients who attended a University Hospital in João Pessoa between February 2018 and January 2022 were included in this study. Regarding the urine samples included in our study, all isolates were obtained as part of routine laboratory diagnostics. The service follows a standard criterion for identifying and storing isolates, which is based on a quantified bacterial load of ≥ 100,000 CFU/mL. This threshold is widely recognized as indicative of a clinically significant infection. Therefore, all urine isolates analyzed in our study were obtained from patients whose samples met this criterion, supporting their association with urinary tract infection (UTI). Isolates previously identified as *S. agalactiae* were inoculated into Brain Heart Infusion (BHI) broth (Kasvi, Parana, Brazil) containing 10% glycerol (Labsynth, São Paulo, Brazil) and stored at −80 °C. Identification at the species level was confirmed by gene *cfb* amplification [15] or by matrix-assisted laser desorption/ionization time-of-flight mass spectrometry using Biotyper 3.1 (Bruker Daltonics, Bremen, Germany). This study was approved by the Hospital Universitário Lauro Wanderley-HULW Ethical Committee (CAAE 02144718.0.3001.5183, statement code 3.155.051).

### 2.2. Serotyping

The capsular type of each isolate was determined using multiplex PCR [16], and the isolates selected for whole-genome sequencing were confirmed using the GBS Typer pipeline v1.0.12 [17].

### 2.3. Antimicrobial Susceptibility Tests

The isolates were tested for susceptibility to penicillin, erythromycin, clindamycin, chloramphenicol, levofloxacin, tetracycline, vancomycin, and linezolid using the disc diffusion method (CECON, São Paulo, Brazil). The minimum inhibitory concentration (MIC) of penicillin was determined using the E-test gradient strip method (Biomerieux, Marcy-L’Etoile, France) and evaluated according to the CLSI interpretive criteria [18].

### 2.4. Statistical Analysis

The chi-square test for the distribution of serotypes among susceptibility profiles was carried out using SPSS 20.0 for Mac OS (IBM Corporation, Armonk, NY, USA) with a significance of 95%.

### 2.5. Analysis of Clonality

Macrorestriction of chromosomal DNA followed by pulsed-field gel electrophoresis (PFGE) was performed as previously described with some modifications [19]. The run conditions were a 6 V/cm gradient, 23 h, 5 s initial switch time, and 35 s final switch time, using the CHEF Mapper system (Bio-Rad, Hercules, CA, USA). The gels were dyed with SYBR Safe DNA Gel Stain (Thermo Fisher Scientific, Waltham, MA, USA) and revealed using the ChemiDoc MP Imaging System (Bio-Rad, Hercules, CA, USA). The PFGE gel image was analyzed using Bionumerics software v. 7.6 (Applied Maths, Sint-Martens-Latem, Belgium) with 1.25% tolerance and 0.5% optimization.

### 2.6. Genome Sequencing

Cultured cells were incubated in lysis buffer mutanolysin 1000 U/mL (Sigma–Aldrich, Saint Louis, MO, USA), lysozyme 50 mg/mL (Sigma–Aldrich, Saint Louis, MO, USA) for 1 h at 37 °C, followed by DNA extraction using the commercial kits DNA Power Soil Kit (QIAGEN, Hilden, Germany) and Wizard Genomic DNA Purification (Promega, Madison, WI, USA) according to the manufacturer’s instructions, and quantified by fluorometry using a Qubit (Life Technologies, Carlsbad, CA, USA) or a Quantus fluorometer (Promega, Madison, WI, USA). For short-read sequencing, a DNA library was prepared using a Nextera XT Kit (Illumina Inc., San Diego, CA, USA). Fragment sizes were evaluated using the Fragment Analyzer capillary electrophoresis system (Agilent Technologies, Santa Clara, CA, USA) and paired-end sequencing was performed on an Illumina MiSeq system (Illumina Inc., San Diego, CA, USA) using a 500-cycle V2 kit (Illumina Inc., San Diego, CA, USA). Reads were quality-checked with FastQC and trimmed using Trimmomatic v 0.38 to remove low-quality reads (Phred < 20) and Illumina sequencing adapters. For long-read sequencing, a DNA library was prepared using Rapid Barcoding Kit 96 (SQK-RBK110.96) (Oxford Nanopore Technologies, Oxford, UK). The pooled libraries were loaded into an R9.4.1 flow cell and sequencing was performed using MinION Mk1B (MinKNOW 22.05.5, Guppy 6.1.5). The long reads were trimmed in Galaxy Web (https://usegalaxy.org/ accessed on 5 October 2023) with Filtlong v 0.2.0 under the default settings, and QC was performed using Nanoplot v1.41.0 [20].

### 2.7. Genome Analysis

The reads were assembled using Flye v.2.9.1, with one polishing iteration [20]. Hybrid assemblies were prepared using Polypolish v.0.5.0 [21]. Alternatively, the filtered long reads and trimmed short reads were assembled using Unicycler v.0.5.0 and samtools v.1.15.1 to compare the final assemblies. The graphical fragment assembly files were loaded on Bandage v.0.8.1, and MLST v.2.22.0 was used to determine the sequence types [20].

The genomes were analyzed using CGE service [22], and mass screening of contigs for AMR genes was performed using ABRicate v.1.0.19. BLASTn was performed to identify the main *S. agalactiae* virulence genes, and the CARD RGI database v.3.2.7 was used to identify antimicrobial resistance genes (ARGs) [23]. Pathogenicity island prediction was performed using GIPSy v.1.1.2 [24] and the results were manually curated according to Lannes-Costa et al. [25]. The PBP (PBP1A, PBP2B, and PBP2X) and pilus-coding genomic islands (PI-1A, PI-2A1, PI-2A2, and PI-2B) were determined using the GBS Typer pipeline [17] and manually checked in the hybrid assembled genomes.

The sequenced genomes were assembled and annotated for genome phylogeny using Prokka v. 1.14.6 and compared with Roary v.3.13.0 [20]. The reference genomes NGBS128, 2603V/R, GBS85147, BM110, and NEM316, with accession numbers NZ_CP012480, AE009948, CP010319, NZ_LT714196, and NC_004368.1, respectively, were annotated and loaded for comparison. A core genome phylogenetic tree was generated using RAxML v.8.2.12 to infer the maximum likelihood [20], and iTOL v.6 [26] was used to visualize the phylogenetic tree. The Jaccard index was calculated as follows: J = number of unique genes/number of core genes.

## 3. Results

### 3.1. S. agalactiae Clonal Relationships, Serotype Distributions and Antibiotic Susceptibilities

**Figure 1:**
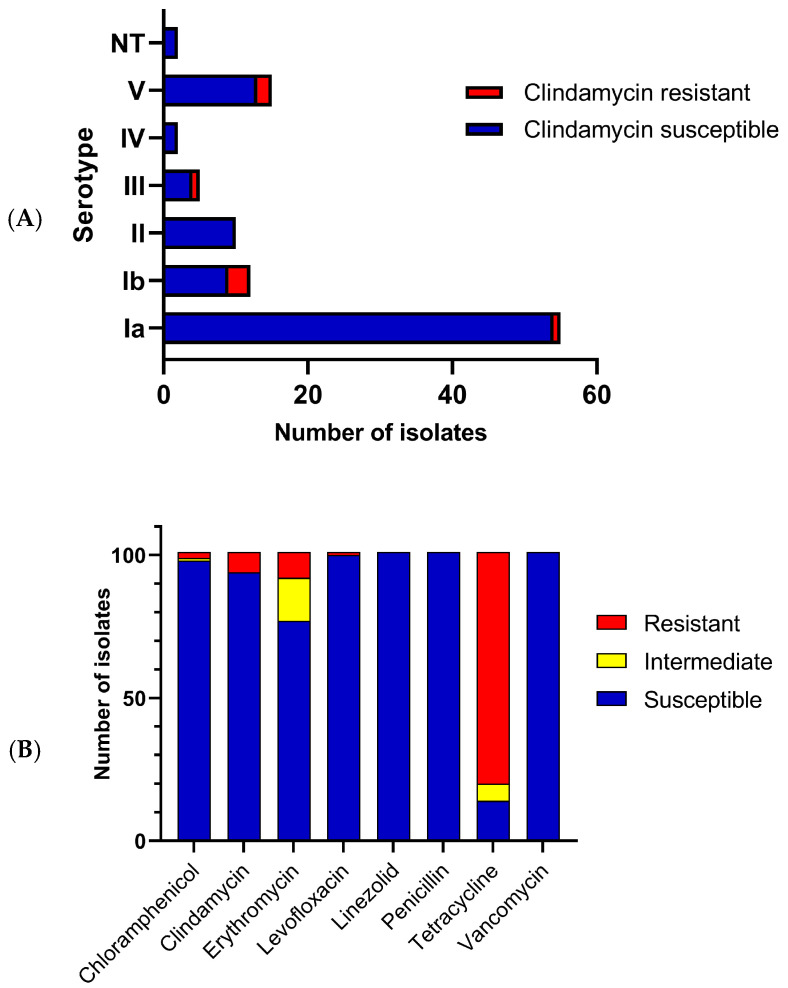
Serotype distribution and antibiotic susceptibility of *Streptococcus agalactiae* from patients from João Pessoa—PB, Brazil, between 2018 and 2022. (**A**) Susceptibility to clindamycin and serotype distribution; (**B**) Antibiotics susceptibility occurrence.

Of the 101 GBS isolates included in this study, 53 were obtained from vaginal or anovaginal colonization of pregnant women and 48 from infection sites (Supplementary File Table S1). PFGE analysis clustered the isolates into 58 pulsotypes. The highest strain similarity was observed in pulsotype H3, with 80.7% similarity, divided into 11 sub-pulsotypes, including 14 strains from serotype Ia and one from serotype Ib (Figure S1). The most prevalent serotype was Ia (n = 55, 54.45%), followed by V (n = 14, 13.86%), Ib (n = 12, 11.88%), II (n = 10, 9.90%), III (n = 5, 4.95%), and IV (n = 3, 2.97%). Two isolates were considered non-typeable by PCR (Figure 1).

All the isolates were susceptible to linezolid, penicillin, and vancomycin (Figure 1). Penicillin MIC values ranged from 0.023–0.094 µg/mL, with MIC_50_ and MIC_90_ values of 0.047 and 0.064 µg/mL, respectively. Resistance was observed to tetracycline (n = 81, 80.20%), erythromycin (n = 9, 8.90%), clindamycin (n = 7, 6.93%), chloramphenicol (n = 2, 1.98%), and levofloxacin (n = 1, 0.99%). Fifteen isolates (14.85%) showed intermediate resistance to erythromycin, six (5.94%) to tetracycline, and one (0.99%) to chloramphenicol. Clindamycin resistance was more frequent in serotypes Ib, III, and V (*p* = 0.024) (Figure 1). Four isolates (HU04_21, HU19_21, HU35_21, and HU71_21) showed constitutive resistance to macrolides–lincosamide–streptogramin B (MLSb) and three (MA06, HU45_21, and HU73_21) showed the phenotype of resistance to macrolides–lincosamide–streptogramin B induced by erythromycin (inducible MLSb). The serotype V MA06 strain exhibited resistance to more antibiotics, and detailed profiles are available in Supplementary Figure S1.

### 3.2. S. agalactiae Genome Analysis

Fourteen isolates were selected for genome sequencing based on serotype and PFGE diversity: five serotype Ia isolates (HU29_21, HU30_21, HU62_21, MA07, and MA12; pulsotypes H1, H14a, H3h, H3k, and H29, respectively), one serotype Ib isolate (HU60_21) belonging to pulsotype H32b, two serotype II isolates (HU05_19 and HU32_21, pulsotypes H56 and H13, respectively), two serotype III isolates (HU19_21 and HU36_21, pulsotypes H6a and H6b, respectively), two serotype IV isolates (HU13_21 and MA15, pulsotypes H12 and H48b, respectively), and two serotype V isolates (MA01 and MA06, pulsotypes H55 and H28, respectively).

Table 1 shows the presence of virulence genes (*bac*, *bca*, *cfb*, *cspA*, *cylE*, *fbsA*, *fbsB*, *fbsC*, *hylB*, *pbsP*, *rib*, *scpB*, *sip*, and *srr1*), pilus islands, and antibiotic resistance determinants. Analyses of the hybrid genomes revealed that HU62_21 harbored a PBP2B allele with only the V80A substitution compared to the reference strain 2603V/R (AE009948).

Because *S. agalactiae* ST17 lineage is known to be hypervirulent and pathogenicity islands (SagPAI) are already very well described in its clones [25], we searched for complete or partial SagPAI in the isolates of this study, irrespective of the STs found or the clinical specimen (Supplementary File Table S2). SagPAI-10, carrying a copy of the *cfb* gene, was complete in all isolates in this study. SagPAI-1 was also found in all isolates but with varied compositions, leading to different serotypes. Except for the isolates belonging to ST1983 (CC103), all remaining isolates harbored SagPAI-11, which contains the *scpB* and *lmb* genes. On MA06, IS*1548* was detected in the *scpB-lmb* intergenic region. SagPAI-2 was complete in 11 of the 14 isolates sequenced in this study, of which 10 carried the transposon *Tn916* with the *tetM* gene. SagPAI-2 was absent in *S. agalactiae* HU05_19 (ST28), MA12 (ST103), and HU19_21 (ST1983). The remaining PAIs were either absent, partial, or complete (Supplementary File Table S2). Isolate MA15 had some SagPAI-5 ORFs that merged with SagPAI-10. SagPAI-8 was absent only in the HU62_21 isolate but harbored the *rib* gene, which is usually present in this PAI. Fragments of SagPAI-8 were found in other isolates. SagPAI-9 was complete in 11 isolates but presented a truncated VncS sensor kinase ORF in HU36_21 and HU62_21. SagPAI-9 was disrupted in HU29_21 and HU30_21, retaining only one paratox gene.

**Figure 2:**
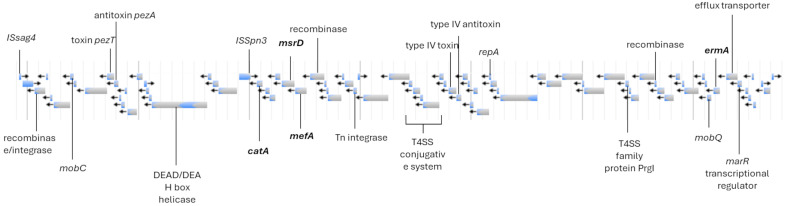
The fragment of MA06 contig 2 (from nucleotide 610,719 to 698,225) shows ARGs (bold) and several relevant genes for mobility with identity of 99.9% and coverage of 86.4% with *S. agalactiae* SA5087 (CP107524).

We searched for acquired resistance genes or mutations related to antibiotic resistance in the genome. Isolate MA06 was the only isolate that presented substitutions in DNA Gyrase GyrA (S81L), topoisomerase IV ParC (S79F, N638S, E640D, R691S, and S739F), and ParE (L1M, H225Y, P356S, and I506V). MA06 also harbored the resistance genes *ermA* (codifying ribosomal methylase), *catA* (chloramphenicol acetyltransferase modifying enzyme), *mefA* (codifying efflux pump), and *msrD* (codifying efflux pump) within a ~100 kbp region, harboring integrases, recombinases, and toxin/antitoxin systems (Figure 2).

**Figure 3:**
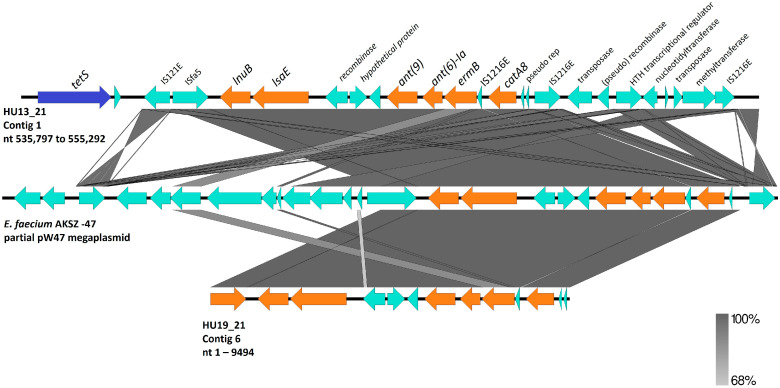
Alignment of a fragment of contig 1 (535,797 to 555,292) from *S. agalactiae* HU13_21 (**top**) and a fragment of contig 6 (1–9494) from *S. agalactiae* HU19_21 (**bottom**) to part of the plasmid pW47 from *E. faecium* AKSZ-47 (accession number CP096051.1). The dark blue arrow shows the *tetS* gene, the orange arrows show resistance genes, and the light blue arrows indicate other ORFs. Nt, nucleotides.

Strains HU13_21 and MA15 belonged to ST196 (CC459) and serotype IV, and harbored several resistance genes. Leader peptide sequences one and two of *ermA* and *ermB* genes were missing in both isolates. Additionally, MA15 harbors a truncated *msrD* gene that is located downstream of *mefA*. We found a 19,495 bp region containing the ARGs in HU13_21 flanked by *IS1216* genes downstream of *tetS* (Figure 3). A deletion at position 542 was observed in the *lnuB* sequence of HU13_21, which resulted in a truncated protein.

Isolates HU36_21 and HU19_21 belonged to a new sequence type (ST1983). Although they belonged to the same lineage, HU19_21 showed resistance to several antibiotics because of the presence of a region with high identity to the pW47 plasmid of *Enterococcus faecium* (Figure 3), which was integrated into the chromosome, harboring *lnu*, *lsaE*, *ant(6)-Ia* (aminoglycoside nucleotidyltransferase modifying enzyme), and *ermB*. HU19_21 also harbored the resistance genes *tetS* and *catA8*. These isolates had the lowest numbers of virulence genes.

### 3.3. S. agalactiae Genome Phylogeny

**Figure 4:**
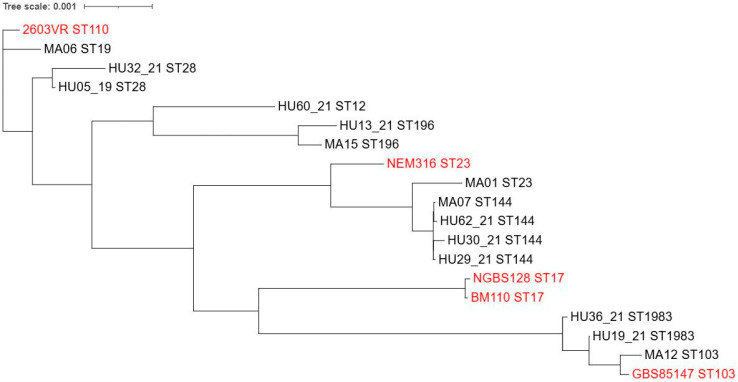
Genome phylogeny of 14 *Streptococcus agalactiae* isolates from human clinical specimens in João Pessoa—PB, Brazil, between 2018 and 2022. *S. agalactiae* 2603V/R (ST110. clinical isolate), *S. agalactiae* NGBS128 (ST17, neonatal disease), *S. agalactiae* BM110 (ST17), *S. agalactiae* GBS85147 (ST103, oropharynx in Brazil), and *S. agalactiae* NGBS128 were included in the analysis as reference strains for comparison using the following accession numbers NZ_CP012480, AE009948, CP010319, NZ_LT714196, and NC_004368.1, respectively. Tree scale: 0.001.

A maximum likelihood phylogenetic tree was generated using the *S. agalactiae* isolates from this study and the reference strains (Figure 4).

## 4. Discussion

Beta-lactams, such as penicillin and amoxicillin, are the first-line treatment for IAP and GBS infections. However, reduced susceptibility due to PBP2X amino acid substitutions is an emerging concern [10,27] and substitutions in PBP1A and PBP2B have been reported, including V80A in PBP2B, which was previously observed in isolates with a penicillin MIC of 1 µg/mL [27]. Notably, HU62_21 exhibited a penicillin MIC of 0.047 µg/mL, suggesting that V80A alone may be unable to significantly reduce susceptibility. According to the CLSI guidelines, GBS are considered susceptible if the penicillin MIC is ≤ 0.12 µg/mL [18]. The observed MIC_90_ values in this study indicate that penicillin remains highly effective in northeastern Brazil.

Resistance rates to macrolides are increasing in Brazil [11], although they are still lower than those in Europe [12] and Asia [28]. Two main mechanisms drive resistance to macrolides in GBS: (1) enzymatic modification of the 23S ribosome via *ermA*/*B* genes, conferring resistance to macrolides, lincosamides, and streptogramin B (MLSb); and (2) macrolide efflux pumps (*mef* and *msr*), which are related to the M phenotype (resistance to macrolides alone) [12]. The *erm* genes are preceded by regulatory peptides (ErmAL1 and ErmAL2 or ErmBL1 and ErmBL2) [29], and the missing leader peptides in MA15 and HU13_21 could affect the induction of *erm* genes expression. However, the mechanism by which these leader peptides interact to induce *erm* expression is poorly understood. *mefA* is widely used as a marker of macrolide resistance in streptococci. However, *msrD* is required for the M phenotype [30], and the absence of functional MsrD in MA15 probably contributes to macrolide susceptibility.

Clindamycin is a suitable second-line IAP and an important therapy for invasive diseases. Resistance to clindamycin is primarily associated with MLSb or the infrequent L phenotype (resistance only to lincosamides), driven by *lnuB*, *lsaC*, or *lsaE* [10]. In our study population, all clindamycin-resistant isolates were resistant to erythromycin. Genome analysis revealed the presence of *ermA*, *ermB*, *lnuB*, and *lsaE.* LnuB is a nucleotidyltransferase and is the main genetic determinant associated with the L phenotype in GBS. LsaE is a member of the efflux pump superfamily of ABC transporters [31]. *lsaE* is usually reported in tandem upstream of *lnuB* [32], and it is not yet established whether *lsaE* expression alone could result in resistance to clindamycin.

Over the last few decades, the emergence of serotype IV in North America has been driven by CC459 strains that are resistant to macrolides and lincosamides [33]. However, in this study, the two IV/ST196/CC459 isolates did not exhibit these profiles, despite the presence of several ARGs. A study comparing the phenotype–genotype of clinical isolates showed high discordance, especially among macrolides and tetracycline in streptococci; 8.2% of the results were discordant, mainly due to phenotypic susceptibility to the presence of ARGs [34]. Furthermore, the genetic context of the ARGs in these isolates is highly complex.

The estimated prevalence of fluoroquinolone non-susceptible isolates is low, with a 2.3% recovery rate of invasive GBS isolates in the USA. The substitutions S81L in GyrA and S79F in ParC found in MA06 are key mutations that result in high resistance to ciprofloxacin and levofloxacin, respectively [10].

Resistance to tetracycline is a feature of human-adapted GBS, primarily driven by *tetM* as part of the transposon *Tn916* [35]. *tetO* and *tetS*, which are less frequent in this species [36], were also observed. Although we did not determine aminoglycoside susceptibility, genome sequencing revealed two isolates harboring the *ant(6)-Ia* gene.

CC23 was related to pulsotype H3, the most frequent pulsotype in our population; CC23 is highly associated with serotype Ia [37]. However, one ST23 isolate (MA01) exhibited an uncommon serotype (V) for CC23, indicating a possible capsular switching event. The potential for capsular switching in GBS has been reported; however, the selective pressures driving this phenomenon remain unclear [12]. The capsular polysaccharide of GBS is a major virulence factor with ten serotypes identified (Ia, Ib, II, III, IV, V, VI, VII, VIII, and IX). The regional prevalence of serotypes is not uniform [37]; serotype Ia is the most prevalent colonizer in pregnant women, and serotype III is associated with at least half of the invasive diseases in newborns. Serotype V is the most common serotype in elderly patients [1,3]. Recently, a trend of serotypes V and Ib was reported in Brazil [11]. In Portugal, these serotypes were associated with increased antibiotic resistance [12]. Serotypes V and Ib were the second and third most prevalent serotypes, respectively, in our population, and there was a significant association between clindamycin resistance and these serotypes. However, all serotypes observed in our population were covered by capsular vaccine formulations in development, including Ia, Ib, II, III, IV, and V [2], except for two non-typeable isolates. Considering this, introducing a hexavalent capsular vaccine formulation could have high coverage in northeastern Brazil, with a low risk of serotype replacement.

Whole-genome sequencing has revealed the repertoire of additional virulence factors involved in human colonization and pathogenesis. *fbsA*/*B*/*C* and *srr1*/*2* are associated with epithelial cell adhesion. *cfb*, *cylE*, and *bca* genes are related to tissue and cell injury. *pbsP* and *hylB* genes have been implicated in tissue invasion. Genes such as *cspA*, *hylB*, *sip*, *rib*, and *bac* impair the immune response mechanisms. In addition, *lmb* encodes a laminin protein that binds to human laminin, and *scpB* encodes the serine protease C5a peptidase [4,38].

The development of a vaccine based on the N-terminal residues of alpha-like proteins is ongoing [39]. Another remarkable finding in this study was the high prevalence of *bca* and *rib* genes, which encode surface alpha-like proteins. Among the 14 sequenced isolates, at least one alpha-like protein was found in each isolate, suggesting possible high coverage of the vaccine formulation.

Additionally, GBS pili are associated with persistent colonization, virulence, and biofilm formation [9]. The genes for synthesis and assembly are clustered on pilus islands (PI) [40]. Previously, PI-2A was found to be highly associated with biofilm formation, and was most frequently observed in our population. PI-2B in association with PI-1 is prevalent in hypervirulent strains, such as CC17, but PI-2B alone is a feature of bovine-adapted strains [41].

MA06 contains IS*1548* in the *scpB-lmb* intergenic region. This integration was previously associated with the higher expression and binding ability of Lmb, suggesting that IS*1548* could upregulate the *lmb* gene [7]. Furthermore, the presence of *pht* in the same region is associated with increased adhesion and invasion [42].

We observed one isolate, Ia/ST103, the respiratory tract. Indeed, the ability to survive under aerobic stress conditions and colonize the respiratory tract seems to be a feature of Ia/ST103 strains [43]. In addition, data from Brazil suggest that specific lineages found in humans and cattle, such as CC103, could significantly impact the Brazilian population [35].

Finally, we report two ST1983 strains (HU19_21 and HU36_21), a single-locus variant of ST651 related to pig offal in Chinese markets [44]. The genomic region containing the resistance genes in HU19_21 was previously reported in a plasmid from a highly resistant *E. faecium* strain isolated from piglets. Structural analysis suggests that horizontal gene transfer is the primary mechanism for dissemination among Gram-positive bacteria [45]. Tetracycline resistance is rare among CCs specialized in cattle but is widespread among the host generalist CC103 [35]. The ST1983 isolates exhibited a tetracycline resistance phenotype; however, they did not harbor the *scpB* and *lmb* genes, which are highly associated with human isolates [7]. Additionally, HU19_21 lacked the *pbsP* gene. Another noteworthy aspect is the absence of PI-1 and presence of PI-2B in both isolates, a feature of bovine strains [41]. The Sag-PAI profiles of the ST1983 isolates were the most distinct, and phylogenetic analysis showed that these two isolates were more distantly related to the most human-adapted strains. A limitation of this study is that there were no patient data correlating virulence traits with treatment outcomes.

GBS has a small core genome that can be associated with high plasticity and diverse host ranges [10]. The hybrid assembly of genomes allowed us to identify the genetic background that could affect adaptation capacity. In bovine hosts, programs to control mastitis have almost eradicated some GBS lineages, and the re-emergence of GBS in cattle is related to lineages from human hosts [46]. This demonstrates that eliminating a specialized lineage could result in the emergence of multi-host lineages, emphasizing the need for continuous surveillance.

## 5. Conclusions

In conclusion, there is a high genetic diversity and prevalence of virulence genes among GBS strains in northeast Brazil. Serotypes V and Ib might be increasing along with clindamycin resistance, but the vaccines under development could have high coverage in our population. Finally, the hybrid assembly of genomes revealed GBS adaptability and virulence, especially of strain ST1983, which suggests a multi-host genetic background.

## Figures and Tables

**Table 1:** Epidemiologic, resistome, and virulome features of 14 *Streptococcus agalactiae* isolates from human clinical specimens in João Pessoa—PB, Brazil, between 2018 and 2022.

Isolate	Source	ST	CC	Resistance Genes	Virulence Genes	Pilus Island	Access Number
HU05_19	Urine	28	19	*tetO*	*bca*, *cfb*, *cspA*, *cylE*, *fbsA*, *fbsB*, *fbsC*, *hylB*, *lmb*, *pbsP*, *rib*, *scpB*, *sip*	PI-1 PI-2A2	CP134689
HU32_21	Urine	28	19	*tetM* ^1^	*bca*, *cfb*, *cspA*, *cylE*, *fbsA*, *fbsB*, *fbsC*, *hylB*, *lmb*, *pbsP*, *rib*, *scpB*, *sip*, *srr1*	PI-1 PI2A2	CP135102
MA06	Urine	19	19	*catA*, *ermA*, *gyrA* (S81L), *mefA*, *msrD parC* (S79F), *tetM*	*bca*, *cfb*, *cspA*, *cylE*, *fbsA*, *fbsB*, *fbsC*, *hylB*, *lmb*, *pbsP*, *rib*, *scpB*, *sip*, *srr1*	PI-2A2	JASKHU000000000
HU13_21	Anovaginal	196	459	*ant(6)-Ia*, *cat*, *ermB *^2^, *lnuB *^3^, *lsaE*, *tetS*	*bca*, *cfb*, *cspA*, *cylE*, *fbsA*, *fbsB*, *fbsC*, *hylB*, *lmb*, *pbsP*, *rib*, *scpB*, *sip*, *srr1*	PI-1	CP135101
MA15	Vaginal	196	459	*ermA ^2^*, *mefA*, *msrD ^3^*, *tetM*	*bca*, *cfb*, *cspA*, *cylE*, *fbsA*, *fbsB*, *fbsC*, *hylB*, *lmb*, *pbsP*, *rib*, *scpB*, *sip*, *srr1*	PI-1	CP135104
HU19_21	Anovaginal	1983	103	*ant(6)-Ia*, *catA8*, *ermB*, *lnu*, *lsaE*, *tetS*	*bca*, *cfb*, *cspA*, *cylE*, *fbsA*, *fbsB*, *fbsC*, *hylB*, *rib*, *sip*, *srr1*	PI-2B	JASKHP000000000
HU36_21	Anovaginal	1983	103	*tetM*	*bca*, *cfb*, *cspA*, *cylE*, *fbsA*, *fbsB*, *fbsC*, *hylB*, *pbsP*, *rib*, *sip*, *srr1*	PI-2B	CP135103
MA12	Tracheal	103	103		*bca*, *cfb*, *cspA*, *cylE*, *fbsA*, *fbsB*, *fbsC*, *hylB*, *pbsP*, *rib*, *scpB*, *sip*, *srr1*	PI-2B	CP143101
HU60_21	Anovaginal	12	12	*tetM*	*bac*, *bca*, *cfb*, *cspA*, *cylE*, *fbsA*, *fbsB*, *fbsC*, *hylB*, *lmb*, *pbsP*, *rib*, *scpB*, *sip*, *srr1*	PI-1 PI-2A2	JAWIIQ000000000
MA01	Vaginal	23	23	*tetM*	*bca*, *cfb*, *cspA*, *cylE*, *fbsA*, *fbsB*, *fbsC*, *hylB*, *lmb*, *pbsP*, *rib*, *scpB*, *sip*, *srr1*	PI-2A1	JASKHT000000000
MA07	Vaginal	144	23	*tetM*	*bca*, *cfb*, *cspA*, *cylE*, *fbsA*, *fbsB*, *fbsC*, *hylB*, *lmb*, *pbsP*, *rib*, *scpB sip*, *srr1*	PI-2A1	CP134688
HU29_21	Urine	144	23	*tetM*	*bca*, *cfb*, *cspA*, *cylE*, *fbsA*, *fbsB*, *fbsC*, *hylB*, *lmb*, *pbsP*, *rib*, *scpB*, *sip*, *srr1*	PI-2A1	JAZGQP000000000
HU30_21	Urine	144	23	*mefA*, *msrD*, *tetM*	*bca*, *cfb*, *cspA*, *cylE*, *fbsA*, *fbsB*, *fbsC*, *hylB*, *lmb*, *pbsP*, *rib*, *scpB*, *sip*, *srr1*	PI-2A1	JAZGQQ000000000
HU62_21	Anovaginal	144	23	*tetM*	*bca*, *cfb*, *cspA*, *cylE*, *fbsA*, *fbsB*, *fbsC*, *hylB*, *lmb*, *pbsP*, *rib*, *scpB*, *sip*, *srr1*	PI-2A1	JASKHS000000000

^1^ Premature stop codon; ^2^ missing signal peptides; ^3^ deletions in sequence. ST = sequence type; CC = clonal complex.

## Data Availability

The original contributions presented in this study are included in the article/Supplementary Materials. Further inquiries can be directed to the corresponding authors.

## Supplementary Materials

The following supporting information can be downloaded at: https://www.mdpi.com/article/10.3390/pathogens14030292/s1, Table S1: Genome data of each isolate; Table S2: *Streptococcus agalactiae* Pathogenicity Island (SagPAI) screening of each isolate; Figure S1: Genetic similarity, pulsotype, serotype, clinical specimen, and susceptibility profiles of 101 *S. agalactiae* isolates.
