# Supplementary material for: Genomic Diversity, Virulome, and Resistome of Streptococcus agalactiae in Northeastern Brazil: Are Multi-Host Adapted Strains Rising?
Source: Pathogens. 2025 Mar 17;14(3):292. doi: 10.3390/pathogens14030292 (PMC11945199; doi:10.3390/pathogens14030292)
Supplement: Supplementary file 1 [file pathogens-14-00292-s001.zip › Table S1.pdf]

Table S1. Genome data of each isolate.

Isolate	Number of Contigs	Length (total)	N50	% GC	Coverage Nanopore	Coverage Illumina
HU05_19	1	2,166,988	2,166,988	35.6	73 x	73 x
HU13_21	1	2,098,259	2,098,259	35.4	40 x	82 x
HU19_21	16	2,083,522	677,260	35.5	23 x	101 x
HU29_21	1	2,157,656	9,602	36	109x	228x
HU30_21	1	2,028,488	3,120	36	60x	179x
HU32_21	1	2,071,976	2,071,976	35.4	35 x	83 x
HU36_21	1	2,130,023	2,130,023	35.4	45 x	82 x
HU60_21	2	2,070,240	2,064,409	35.4	53 x	75 x
HU62_21	3	2,056,893	1,106,711	35.5	14 x	88 x
MA01	3	2,103,843	2,084,689	35.5	37 x	91 x
MA06	44	2,188,773	698,225	35.5	19 x	82 x
MA07	1	2,055,223	2,055,223	35.5	40 x	75 x
MA12	1	2,275,014	14,061	37	110x	73x
MA15	1	2,148,820	2,148,820	35.7	80 x	75 x
