# Supplementary figures and images for: Genomic Diversity, Virulome, and Resistome of Streptococcus agalactiae in Northeastern Brazil: Are Multi-Host Adapted Strains Rising?
Source: Pathogens. 2025 Mar 17;14(3):292. doi: 10.3390/pathogens14030292 (PMC11945199; doi:10.3390/pathogens14030292)

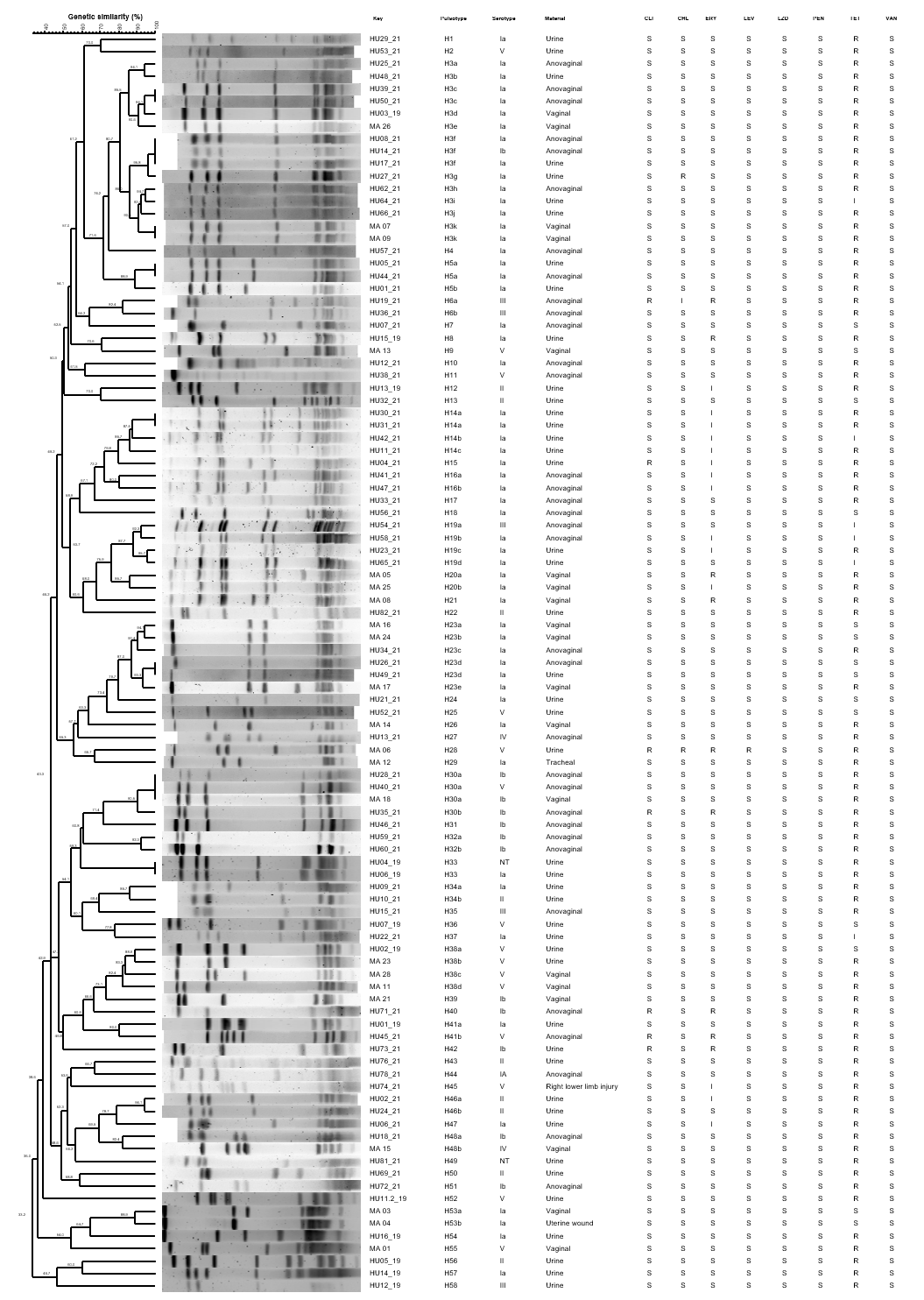

Supplement: Supplementary file 1 [file pathogens-14-00292-s001.zip › Figure S1.tif]
